# EGFR inhibitor C225 increases the radiosensitivity of human lung squamous cancer cells

**DOI:** 10.1186/1475-2867-10-39

**Published:** 2010-10-23

**Authors:** Yingdong Zhang, Junjie Wang, Feng Liu, Zhenyu You, Ruijie Yang, Yong Zhao

**Affiliations:** 1 Department of Radiation Oncology, Cancer center, Peking University Third Hospital, Beijing, P.R. China 100191; 2 Transplantation Biology Research Division, State Key Laboratory of Biomembrane and Membrane Biotechnology, Institute of Zoology, Chinese Academy of Sciences, Beijing, P.R. China 100101

## Abstract

**Background:**

The purpose of the present study is to investigate the direct biological effects of the epidermal growth factor receptor (EGFR) inhibitor C225 on the radiosensitivity of human lung squamous cancer cell-H520. H520 cells were treated with different dosage of ^60^Co γ ray irradiation (1.953 Gy/min) in the presence or absence of C225. The cellular proliferation, colony forming capacity, apoptosis, the cell cycle distribution as well as caspase-3 were analyzed in vitro.

**Results:**

We found that C225 treatment significantly increased radiosensitivity of H-520 cells to irradiation, and led to cell cycle arrest in G_1 _phase, whereas ^60^Co γ ray irradiation mainly caused G_2 _phase arrest. H-520 cells thus displayed both the G_1 _and G_2 _phase arrest upon treatment with C225 in combination with ^60^Co γ ray irradiation. Moreover, C225 treatment significantly increased the apoptosis percentage of H-520 cells (13.91% ± 1.88%) compared with the control group (5.75% ± 0.64%, P < 0.05).

**Conclusion:**

In this regard, C225 treatment may make H-520 cells more sensitive to irradiation through the enhancement of caspase-3 mediated tumor cell apoptosis and cell cycle arrest.

## Introduction

It is well known that many non-small cell lung cancer (NSCLC) cells over-express membrane surface epidermal growth factor receptor (EGFR) [1-7]. EGFR activation led to cell proliferation, angiogenesis and apoptosis inhibitory cytokines related phosphorylation and activation of cell signal pathway[8,9]. EGFR monoclonal antibody cetuximab (C225, Erbitux, Merck KGaA, Germany) inhibits tumor growth by directly impeding the EGFR ligands EGF and transforming growth factor-α (TGF-α) combination of the above-mentioned cell block access [9-16]. In the in vitro studies, C225 combined with radiotherapy or chemotherapy inhibits the growth of head and neck squamous carcinoma cells dramatically. Furthermore, in the clinical treatment of head and neck squamous cell carcinoma [17,18], C225 combined with radiotherapy has also made a fantastic therapeutic effect [14,15]. At present, the clinical trials of C225 combined with radiotherapy and radiotherapy alone for patients with advanced NSCLC are under way[18], no clear conclusion has been drawn. In this experiment H-520 cell proliferation, apoptosis and cell cycle distribution were detected after treating with C225 combined with radiotherapy.

## Materials and methods

### Cell lines and culture

The human non-small cell lung cancer cell line H-520 was purchased from Institute of Basic Medical Science, Peking Union Medical College (Beijing, China). H-520 cells were maintained in DMEM medium which was composed of 10% fetal calf serum, 1% penicillin and streptomycin (penicillin 100 U/ml, streptomycin 100 mg/ml), and 1% glutamine. Cells were cultured in 37°C incubator with 5% CO_2_.

### Determination of EGFR expression in H-520 cells by flow cytometry

Exponentially growing H-520 cells were adjusted to 5 × 10^6^/ml and incubated with mouse-against human EGFR antibody for 30 min at 4°C. After washing with PBS twice, the cells were treated with FITC labeled goat-anti-mouse IgG for 30 min. The EGFR expression was detected by flow cytometry.

### Cell treatment with C225 and irradiation

H520 cells were pre-cultured with 40 nM C225 for 12 h, these cells and control cells then received a single dose of γ ray irradiation from a ^60^Co source (Peking University Health Science Center, China). The irradiation rate is 1.953 Gy/min.

### Cell growth analysis by methyl thiazolyl tetrazolium (MTT) assay

Cell proliferation was determined by assessing the mitochondrial reduction of MTT. Cells were plated at 1 × 10^3 ^cells/well in 96-well plates containing 200 μl growth medium and allowed to attach for 24 h. The medium was removed. Blank control and C225 (0.008, 0.04, 0.2, 1, 5, 25, 125 and 625 nM) groups were prepared and cells were incubated for 0, 12, 24, 48, 72 and 120 h. At harvest, the medium was removed from the appropriate wells, replaced with 50 μl MTT solution (2.5 mg/ml), and incubated for 4 h at 37°C. After incubation, the MTT solution was carefully aspirated and replaced with 150 ul DMSO. Cell growth was analyzed on a plate reader by using SoftMax program (Molecular Devices Corp., Menlo Park, CA). Experiments were performed in quadruplicate and repeated at least 3 times. Inhibition ratio (%) = (1-A190 of testing group/A190 of the control group) × 100%.

### Colony formation

The cells in exponential growth were digested with trypsin into single cell suspension. Cells were seeded into 100 mm culture plates at various dilutions. Cells were distributed evenly in 10 ml medium and maintained in incubator at 37°C for 14 days. Then the cells were fixed with methanol and stained with Gimsa. The colonies with more than 50 cells were counted. The plating efficiency (PE) was calculated as the formula: PE = number of colonies/number of seeded cells × 100%. Results were shown as the mean value of quadruplicate samples in each dose.

### Dose-survival curve and sensitization enhancement ratio (**SER**)

The H-520 cells were exposed to ^60^Co γ ray irradiation, with the total dose of 100, 200, 400, 600, 800 and 1000 cGy. There are three samples in each dose group. After irradiation, cells were digested and seeded into 100 mm plates at different cell numbers. Then the cells were cultured in 35 mm plates for 14 days, and the formed colonies were counted. The survival fraction (SF) was calculated as the formula: SF = number of colonies/number of seeded cells × PE. The dose-survival curve was fitted based on the one-hit multi-target formula $SF=1-{\left(1-e^{-D/D_{0}}\right)}^{N}$, with cell survival percentage as Y-axis and absorbed dose as X-axis, in which D was the absorbed dose, D_0 _was the mean lethal dose, and N was the extrapolation number. In addition, the N, D_0_, D_q _(quasi-threshold dose) and SERwere calculated based on the dose-survival curve of H-520 cells irradiated with ^60^Co γ ray, SER is defined as following: SER = (D_10 _of the group irradiated with ^60^Co γ ray)/(D_10 _of the group treated with combination of ^60^Co and C225).

### Cell apoptosis determined by Hoechst33258-staining

H-520 cells were separated into three groups: control, C225-treated group and 8 Gy ^60^Co γ ray irradiated group. After incubation for 72 h, the cells were fixed and stained with 100 μg/ml Hoechst 33258 (Beijing Lanbosite Biotech. Co. Beijing, China) for 30 min. Cells morphology were detected under fluorescence microscopy.

### Cell cycle and apoptosis analysis by flow cytometry (FCM)

Cells from the control and C225 groups were exposed to different dose of irradiation (0, 2, 4 and 8 Gy). Cells were harvested at 72 h after irradiation for cell apoptosis analyses by FCM. Cells for cycle analysis (0 and 8 Gy) were harvested at 48 h after irradiation. Each test was performed 3 times. Cells used for cell-cycle tests were stained with propidium iodide. Cells used for apoptosis tests were stained with propidium iodide (PI) and annexin V-FITC and analyzed by fluorescence-activated cell sorting (FACS) using Coulter EPICS and ModFit software (Verity Software House, Topsham, MN).

### The expression levels of Caspase-3 after treatment with C225 or/and irradiation

Four groups were involved in this study: control group, C225 group (final concentration of 40 nM), irradiation group (8 Gy) and combination group (C225 of 40 nM combined with irradiation of 8 Gy). Cells were collected as before and 0.5 μL Caspase-3 specific antibody were added to each tube, and incubated for 15 min away from light after mixing the cell suspension. Cell suspension were adjusted to 300 μl with PBS, and analyzed with FACS.

### Statistical analysis

GraphPad prism software 4.0 was adopted to produce dose-survival curve. The statistics was performed with SPSS11.5 version. Measurement data were presented as mean ± standard deviation (x ± *SD*). The difference between the means of two different groups was analyzed with t-test, while ANOVA was sued for comparing 4 groups of treatment. p < 0.05 was considered significant.

## Results

### High expression of EGFR in H-520 cells

**Figure 1:**
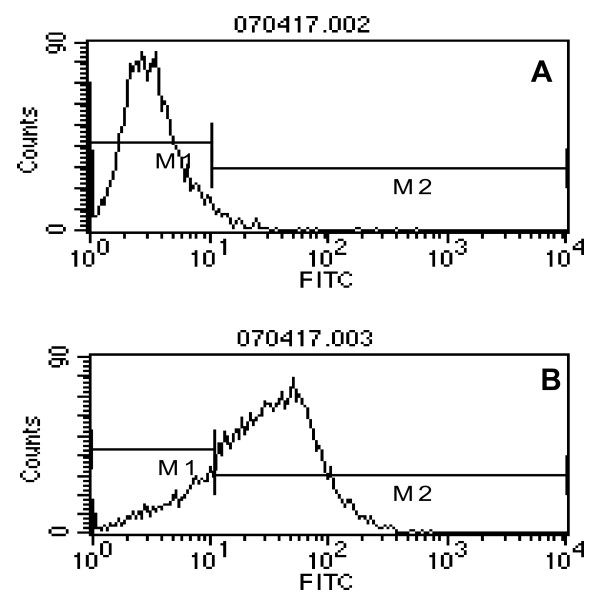
**H-520 cells express high levels of EGFR**. The percentage of EGFR expressing cells in H-520 cells was determined by FACS. The upper panel (A) shows isotype control, which stains only with secondary antibody. The lower panel (B) shows that 82% of the H-520 cells expressed EGFR as stained positively for EGFR mAb. One representative of three experiments was shown.

In order to determine the expression of EGFR in our cell line H520, we firstly performed indirect staining to detect EGFR expression with FACS. The percentage of EGFR expression in normal lung tissue cells is below 10%. However, as shown in Figure 1, 82% of the H-520 cells expressed EGFR.

### Effects of C225 on H-520 cell growth

**Figure 2:**
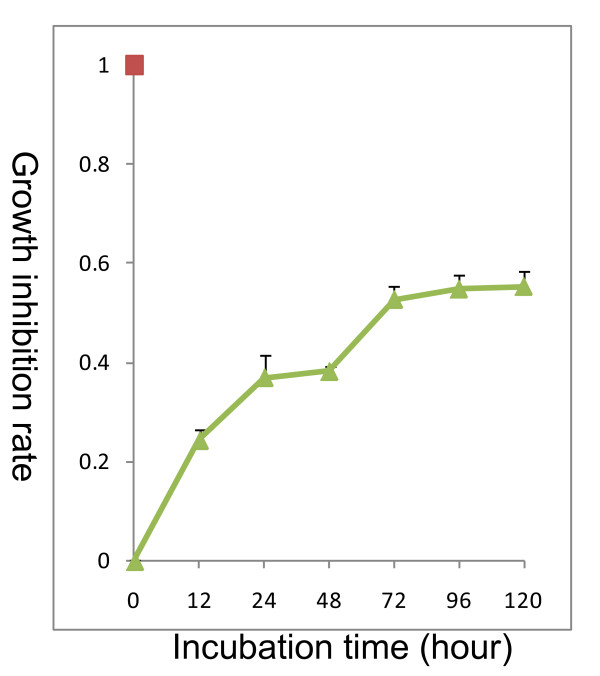
**Growth inhibitory effects of C225 on human non-small cell lung cancer cells**. Using MTT assay, we incubated H-520 cells with 40 nM C225 for different time points, and found that the growth inhibition rate was about 50% 72 hours after 40 nM C225 treatment. Further treatment did not cause higher growth inhibition. One representative of two experiments was shown.

From our previous study, we found the 40 nM C225 treatment showed approximately 50% inhibition of H-520 cell growth. We thus used 40 nM C225 for further analysis. We incubated H-520 cells with 40 nM C225 for different time points, and found that the growth inhibition rate was about 50% 72 hours after 40 nM C225 treatment. Prolonged treatment did not lead to higher growth inhibition, as shown in Figure 2.

### Enhanced radiosensitivity in EGFR inhibitor C225-treated H-520 cells

**Figure 3:**
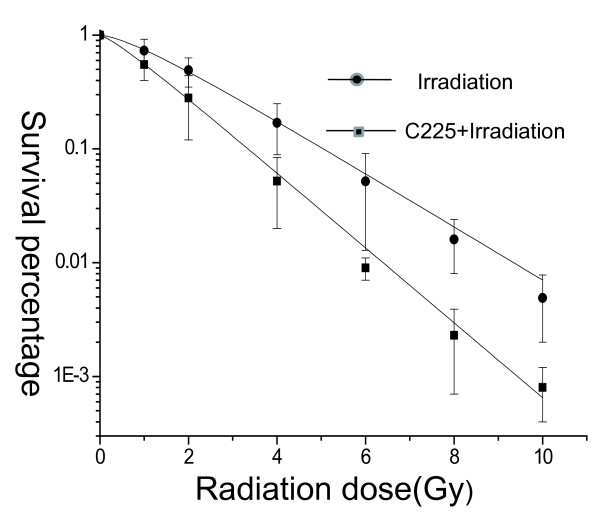
**Radiosensitivity was enhanced by EGFR inhibitor C225**. SER calculation was carried out and we found that treatment with C225 significantly improved the biological effect of irradiation. The radiobiological parameters of C225-treated H-520 cells were D_0 _= 1.32, Dq = 0.19, N = 1.352, while those of irradiated H-520 cells were D_0 _= 1.82, Dq = 0.51, N = 1.81. Data were shown as Mean + SD (N = 4). One representative of three experiments with identical results was shown.

The radiobiological parameters of C225-treated H-520 cells were D_0 _= 1.32 Gy, Dq = 0.19 Gy, N = 1.352, while those of C225-untreated H-520 cells were D_0 _= 1.82 Gy, Dq = 0.51 Gy, N = 1.81. In the present study, the SER was about 1.35, which indicated that treatment with C225 significantly improved the biological effect of irradiation. The results were shown in Figure 3.

### Significantly enhanced cell apoptosis in C225-treated H-520 cells after irradiation

Under fluorescent microscope, it appeared apparently that, compared with irradiation group, C225 treatment together with irradiation significantly induced morphological changes of H-520 cells, including the majority of cells' nuclei was wavy (rippled), cell membrane folded, chromatin condensed, some of which were cracking as the fragments of the nucleus, resulting in the appearance of apoptotic bodies (data not shown).

**Figure 4:**
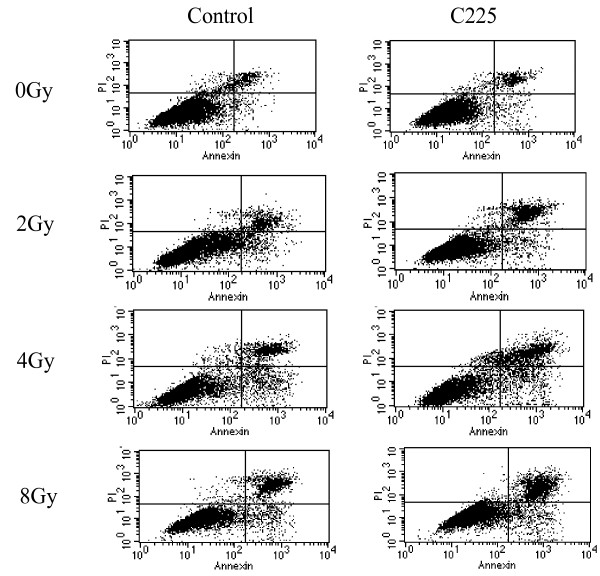
**Apoptosis of H520 cells induced by irradiation and C225**. H520 cells were treated with C225 or/and different doses of irradiation. The apoptosis was determined by flow cytometry. The results were summarized in Table 1. One representative of three experiments with identical results was shown.

The percentage of apoptosis cells was determined by flow cytometry. The apoptosis rate of H-520 cells under conventional culture conditions was about 5%, however C225 rose the percentage of cell apoptosis to 13.91 ± 1.88 (t = 3.59, P < 0.05, Figure 4). The apoptosis percentage of control group irradiated with 2, 4, or 8 Gy γ ray respectively was 13.88 ± 2.38, 24.67 ± 1.77, 30.36 ± 2.81, after treated with C225 coupled with irradiation the apoptosis percentage was 23.33 ± 3.18 (t = 2.16, P < 0.05), 39.01 ± 0.173 (t = 4.74, P < 0.05), and 42.46 ± 4.06 (t = 5.65, P < 0.05), respectively. There was significant difference between C225 and irradiation combination group and irradiation group. The results were summarized in Table 1.

**Table 1:** H-520 cells apoptosis rate

	Apoptosis rate (%, × ± SD)			
Groups	0Gy	2Gy	4Gy	8Gy
Control	5.75 ± 0.64	13.88 ± 2.38	24.67 ± 1.77	30.36 ± 2.81
C225	13.91 ± 1.88^a^	23.33 ± 3.18^b^	39.01 ± 0.73^c^	42.46 ± 4.06^d^

Note: T test for the apoptosis rate of two groups at the same dose,

^a^*P *= 0.02; ^b^*P *= 0.02; ^c^*P *= 0.03; ^d^*P *= 0.04

### C225 arrested the cell cycle of H-520 cells at G_0_/G_1 _phase

**Figure 5:**
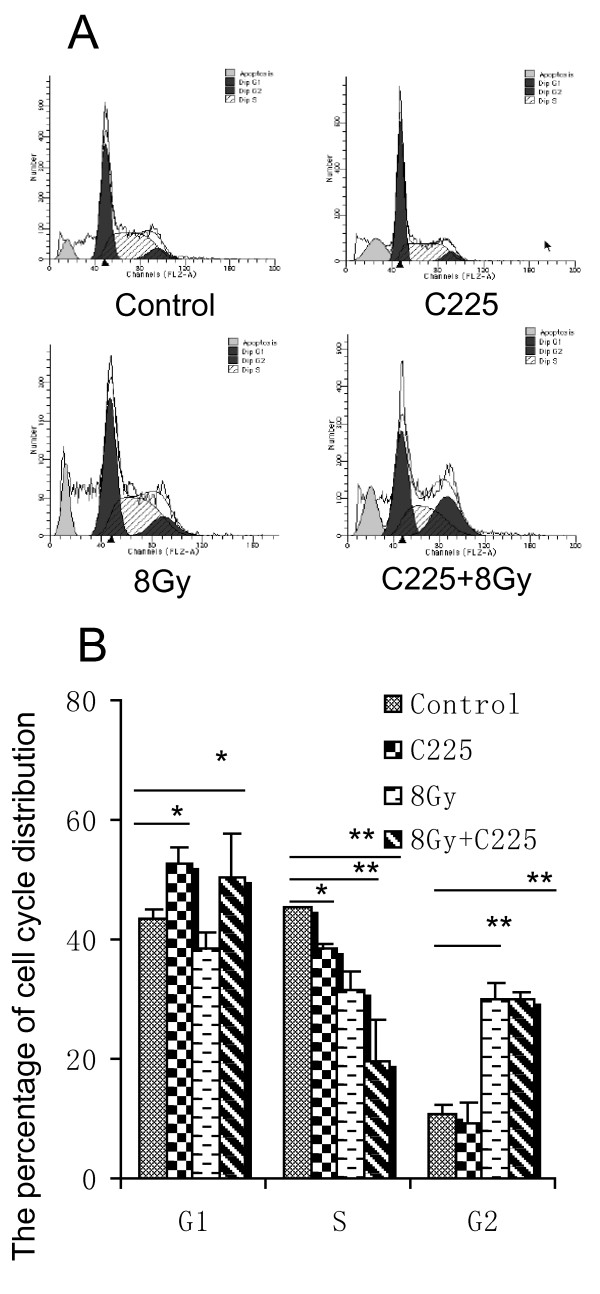
**C225 arrested the cell cycle of H-520 at G_0_/G_1 _phase**. C225 treatment caused H-520 cell cycle arrested at G_0_/G_1 _phase, 52.55 ± 2.67% compared with control cells 43.64 ± 1.54%. In the meantime, γ-ray irradiation arrested the cell cycle at G_2_/M phase from 10.83 ± 1.52% to 29.95 ± 2.6%; C225 treatment together with irradiation led to cell cycle arrest at both G_0_/G_1 _and G_2_/M phase, thus the proportion of S phase reduced from 45.46 ± 0.06% to 21.12 ± 6.84%.

As shown in Figure 5, C225 arrested the cell cycle of H-520 at G_0_/G_1 _phase from 43.64 ± 1.54% to 52.55 ± 2.67% determined by flow cytometry. Meanwhile, γ ray irradiation arrested the cell cycle at G_2_/M phase from 10.83 ± 1.52% to 29.95 ± 2.6%; when C225 coupled with irradiation the cell cycle arrested at both G_0_/G_1 _and G_2_/M phase, meanwhile the proportion of S phase reduced from 45.46 ± 0.06% to 21.12 ± 6.84%.

### C225 increased the ratio of caspase-3 in H520 cells

**Figure 6:**
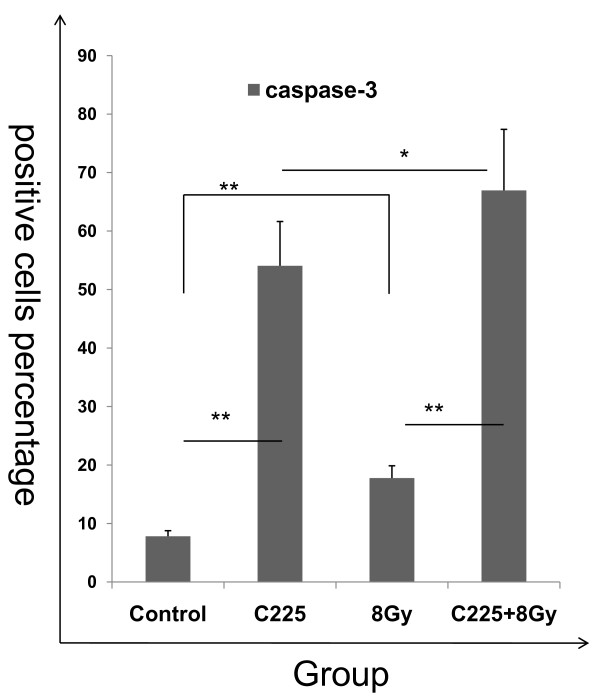
**C225 increased the ratio of caspase-3 positive cells**. C225 can significantly increase the percentage of caspase-3 positive cells (p < 0.05). The percentage of caspase-3-positive cells in control group, C225 group, irradiated group and C225 coupled with irradiation group was 7.8 ± 0.94, 54.1 ± 7.56, 17.7 ± 2.1, 66.9 ± 10.4, respectively.*p < 0.05, **p < 0.01 compared with the indicated groups.

The percentage of caspase-3-positive cells in control group, C225 group, irradiated group and C225 coupled with irradiation group was 7.8 ± 0.94, 54.1 ± 7.56, 17.7 ± 2.1, and 66.9 ± 10.4, respectively. C225 increased the ratio of caspase-3 positive cells significantly (p < 0.05). The results were shown in Figure 6.

## Discussion

The incidence and mortality of lung cancer are high and Lung cancer is the leading cause of cancer-related deaths [19]. Clinical diagnosis of 80-87% of patients with lung cancer is non-small cell lung cancer. Currently, radiotherapy and chemotherapy combined treatment for locally advanced NSCLC is the standard treatment program. One of the characteristics of chemotherapy is based on two kinds of platinum-based drugs in combination with radiation therapy to sequential or simultaneous delivery methods [20]. Though the new chemotherapy drugs and new drug combination has been introduced into the program, conventional radiotherapy has been substituted by three-dimensional stereotactic radiotherapy. Statistics show that over the past two decades, the survival time of patients with NSCLC remains no change. We found that the clinical treatment of the same stages and even the same type of patients using the same treatment, the patient's prognosis are different, it prompt us to speculate that the tumor pathological type may have a great impact on therapy options and prognosis of patients. In recent years, bio-molecular targeting therapy increasingly receives attention. In non-Hodgkin's lymphoma, renal cell cancer, colorectal cancer, non-small cell lung cancer and head and neck squamous cells cancer treatment, bio-targeting drugs have made a good effect [18,21].

Clinically, anti-EGFR monoclonal antibody C225 combined with radiotherapy showed a significant effect on head and neck squamous cell carcinoma, the C225 combined with radiotherapy as the conventional treatment options on head and neck squamous cell carcinoma have been acceptable to the majority of doctors [21,22]. Many biological characteristics of advanced NSCLC are consistent with the head and neck cancer. Squamous cell carcinoma is the majority of NSCLC, so in this experiment we use H-520 cells as research materials to observe the effects of C225 and ^60^Co γ treatment (alone/coupled) on the proliferation, apoptosis and changes in the cell cycle.

In the present study, we used H-520 cells as human non-small cell lung cancer cells. Though it is reported that H-520 cells do not express EGFR[20,23], we detected high level expression of EGFR as determined by flow cytometry. The inconsistency between our results and the published data may be due to the long term cell culture, the selected clone expansion and the other reasons in our labs, which should be determined in the future. The expression of EGFR may make our H-520 cells more sensitive to C225 treatment so that we can study the co-effect of radiation of C225 in this cell line.

Colony forming efficiency analysis is a classical method to evaluate the anti-tumor ability of γ ray irradiation. Through analysis of cell survival curves we found that the N value of C225 combined with radiotherapy is smaller, the curves had no apparent shoulder area, which indicate that the C225 increased the sensitivity of the H-520 cells to ^60^Co γ ray irradiation. At the same time, the D_0 _Value is relatively lower suggesting that the reasonable lower doses of ^60^Co γ ray can also kill tumor cells when coupled with C225. The SER of C225 treatment is about 1.4 which suggested that C225 had radiosensitization effect on the H-520 cells. It has been studied that after irradiation the expression of EGFR and TGF-α increased which enhanced the ability of the tumor cells to repair DNA damage, and rapidly proliferate [24,25]. The C225 which can combine with EGFR not only blocked the secretion of TGF-α and signal transduction but also decreased the expression of EGFR on the cell membrane surface [16]. These impeded the cell survival and proliferation.

The cell-cycle phase is one of the determinative factors of cell radiosensitivity [26]. In the present study we observed C225 arrested the cell cycle at the G0/G1 phase, ^60^Co γ ray irradiation resulted in G_2_/M phase arrest, C225 in combination with ^60^Co γ ray irradiation at the same time led to the cell cycle arrested at G_0_/G_1_, G_2_/M phase. Under normal condition, cell cycle arrests at some point in order to repair damage, when the damage repairs successfully, cell cycle continues, otherwise cells undergo apoptosis. It needs the participation of many growth factors to repair the irradiation induced damage for the cells arrested at G_2_/M phase [27]. When the EGFR pathway is blocked by C225, the repair is difficult to proceed smoothly, the cells will undergo apoptosis. With this match, C225 in combination with irradiation significantly increased the rate of apoptosis.

Caspase-3 plays an important role in the apoptosis process which hydrolytic DNA led cells to apoptosis, caspase-3 positive cells will be apoptotic eventually [28]. We found that C225 increased the number of caspase-3-positive cells significantly, the mechanism may be that C225 against with EGFR blocked the PI3K pathway which reduced Akt phosphorylation (Akt is an important protein molecule in PI3K pathway, it regulates caspase-9, GSK-3β, NF-κB, such as protein phosphorylation and ultimately controls cell survival and apoptosis) and then activated caspase-9 and process of apoptosis using caspase-3 to cut DNA to complete the task of apoptosis.

In summary, C225, which blocks EGFR pathway, inhibited H520 lung squamous carcinoma cell proliferation, and increased the sensitivity of lung squamous cancer cells to irradiation dramatically.

## Competing interests

The authors declare that they have no competing interests.

## Authors' contributions

YDZ carried out cell colony-forming assay, fluorescence- activated cell sorting, flow cytometric analysis, and drafted the manuscript. JJW participated in its design and revised the manuscript. FL performed the statistical analysis. ZYY carried out the irradiation experiment. RJY revised the manuscript critically for important intellectual content and helped to draft the manuscript. YZ supervised experimental work and revised the manuscript. All authors read and approved the final manuscript.
